# Psychological distress among women undergoing *in vitro* fertilization-embryo transfer: A cross-sectional and longitudinal network analysis

**DOI:** 10.3389/fpsyg.2022.1095365

**Published:** 2023-01-05

**Authors:** Liuliu Wu, Lijing Sun, Juan Wang, Yaoyao Sun, Xuan Zhang, Yongqi Huang, Yan’e Lu, Fenglin Cao

**Affiliations:** ^1^Department of Health Psychology, School of Nursing and Rehabilitation, Cheeloo College of Medicine, Shandong University, Jinan, Shandong, China; ^2^Center for Reproductive Medicine, Cheeloo College of Medicine, Shandong University, Jinan, Shandong, China; ^3^Institute of Mental Health, Peking University Sixth Hospital, Beijing, China

**Keywords:** *in vitro* fertilization, depression, anxiety, network analysis, long-term

## Abstract

**Background:**

Women undergoing *in vitro* fertilization-embryo transfer (IVF-ET) treatment were generally found to experience varying degrees of psychological distress across the treatment. Existing studies focused on total scores and diagnostic thresholds to characterize the symptoms’ severity, which might hinder scientific progress in understanding and treating psychological distress.

**Aims:**

We aimed to investigate (a) how depression and anxiety symptoms are interconnected within a network, and (b) the changes of the network (symptom connections and network centralities) over time, in women undergoing *in vitro* fertilization-embryo transfer.

**Methods:**

A 4-wave longitudinal study was designed with 343 eligible women recruited from the Reproductive Medicine Center of a tertiary hospital in China. The network models were created to explore the relationship and changes between psychopathology symptoms both within and across anxiety and depression, with anxiety measured by the Generalized Anxiety Disorder-7 and depression measured by the Patient Health Questionnaire-9. Symptom network analysis was conducted to evaluate network and network properties, network centrality, and bridge centrality, as well as change trajectory network.

**Results:**

For the strength centrality, “inability to control worry” and “worrying too much” were the most central symptoms at T1; however, these symptoms decreased. The centrality of “sadness” and “guilt” tended to increase steadily and became dominant symptoms. For bridge centrality indices, several bridge symptoms were identified separately from T1 to T4: “irritability,” “concentration difficulties,” “nervousness,” and “restlessness;” “guilt” exhibited increased bridge symptoms. Furthermore, the change trajectory network indicated that “suicide ideation” became more closely related to guilt but not to worrying too much over time.

**Conclusion:**

This study provides novel insights into the changes in central features, connections, and bridge symptoms during IVF-ET treatment and identified several bridge symptoms separately at different stages, which could activate the connection between psychopathology symptoms. The results revealed that sense of guilt was associated with worsening psychopathology symptoms, indicating that future psychological interventions should target guilt-related symptoms as a priority.

## Introduction

Infertility is recognized as a global health problem by the World Health Organization, affecting 20–30% of the female population of reproductive age in modern society (Bai et al., 2019b; Vitagliano et al., 2021). In China, approximately 15.5% of reproductive-age women seek treatment for infertility each year (Zhou et al., 2018). *In vitro* fertilization-embryo transfer (IVF-ET) treatment experience is becoming a life crisis for some and may be particularly devastating for women due to infertility itself, intrusive medical treatments, high financial costs, and uncertainty over treatment effects (Peterson et al., 2007; Wichman et al., 2011; Hakimi and Cameron, 2017; Renzi et al., 2020). A growing body of literature shows that infertile women undergoing IVF-ET treatment experience elevated levels of psychological distress, of which anxiety and depression are the most common (Van den Broeck et al., 2010; Gdanska et al., 2017; Bai et al., 2019a) and is associated with significant adverse treatment outcomes (Smeenk et al., 2001; Quant et al., 2013; Aimagambetova et al., 2020).

The most popular framework to investigate depression, anxiety, and their co-occurring symptoms is the common cause theory that the co-occurrence of symptoms is caused by an unobserved common cause (Borsboom, 2008; Schmittmann et al., 2013). Within this context, in studies of infertile women, it has been hypothesized that depression causes symptoms such as hopelessness, sleep disturbances, and fatigue (Hughes and da Silva, 2011; Huang et al., 2019; Kim et al., 2020), whereas anxiety causes symptoms such as worry, restlessness, and fear (Karatas et al., 2010; Cao et al., 2022). Thus, most mental health research in the infertile women field focused on the total scores or frequencies of distress symptoms to represent psychological distress severity (Lakatos et al., 2017; Cui et al., 2021; Liu et al., 2021; Dadhwal et al., 2022). However, this methodology risks obscuring important variations between specific symptoms and the connections among symptoms due to the assumption that these disorders are separate entities in the common cause model. Psychological symptoms are often the result of direct symptom-symptom connections but not of common causes (Cramer et al., 2010). Furthermore, the common cause perspective does not adequately explain the finding that changes in one symptom can predict changes in other symptoms in the following week (Bringmann et al., 2015). In conclusion, the traditional perspective and approach might hinder scientific progress in understanding and treating psychological distress (Boschloo, 2018; Jones et al., 2019).

The recently emerged network approach, on the other hand, describes psychological distress as systems of connected symptoms rather than as reflecting an unobserved cause (Borsboom, 2017). The symptom’s co-occurrence is based on the network modeling of dynamical systems that mutually cause, maintain, and form the basis of symptomatology (Barrat et al., 2008). For example, in the depression of infertile women, hopelessness may be highly correlated with sleep disturbance, not because they have a common cause (depression), but because hopelessness is directly associated with sleep disturbance. One of the advantages of applying network analysis to the study of psychological distress in infertile women undergoing IVF-ET treatment is to offer a visual depiction of the complex connections among symptoms (Beard et al., 2016; Rouquette et al., 2018). From a clinical point of view, network analysis can also identify the most central symptoms within the infertility-related psychopathology network. Resembling the domino effect, the central symptom may have a greater influence on the network because of its high degree of interconnections (Park and Kim, 2020). The identification of central symptoms is critical for clinicians to develop effective intervention programs. Further, the network model emphasizes the crucial role of bridge symptoms which connect two mental disorders, and the activation of these bridge symptoms is likely to result in the development and maintenance of both disorders (Jones et al., 2019). As such, identifying bridge symptoms between depression and anxiety to prevent co-occurrence has substantial implications for mental health improvement among women undergoing IVF-ET. This could be achieved by implementing targeted and prioritized treatment for bridge symptoms, contributing to the control and prevention of the onset of other depression and anxiety symptoms.

Another key issue that needs attention is understanding how depression and anxiety symptoms change over the diverse stages of IVF-ET treatment. IVF-ET involves different stages, including ovulation induction, oocyte retrieval, and embryo transfer, in which patients face various challenges (Gameiro et al., 2016). A prospective United Kingdom study using three time point data found that women undergoing IVF-ET treatment experienced the highest levels of psychological distress at the stage of the pregnancy test (Yong et al., 2000). The systematic review indicated that women experienced elevated anxiety and depressive symptoms during IVF-ET treatment (Verhaak et al., 2007). In a recent Chinese longitudinal study, women showed the highest depression scores in stage T1 (on the first day of entering the cycle) and the highest anxiety scores in stage T2 (the day human chorionic gonadotropin was administered; Liu et al., 2021). However, to our knowledge, no studies have focused on changes in the relationship between depression and anxiety among women during IVF-ET treatment. Thus, it is essential to investigate the interactions and changes in anxiety and depressive symptoms across the entire treatment pathway.

Therefore, network analysis has the potential to obtain a detailed and fine-grained description of these symptom-symptom interactions and their development, which provides more clinically useful insights than the classic model (Rouquette et al., 2018). However, to our knowledge, the available studies have not explored the connections between anxiety and depressive symptoms among women undergoing IVF-ET treatment using network analysis.

In the current study, we aimed to extend the previous literature on three points. First, we characterized the depression and anxiety symptoms network structure in a large longitudinal sample of women undergoing IVF-ET treatment. Second, we used a novel method to identify the central and bridge symptoms within the networks (Jones et al., 2019). Finally, we investigated whether the network changed over the course of treatment in two ways, firstly, by looking at the network structure patterns at each of the four cross-sectional time points separately, and secondly, by exploring the connections between symptoms in a longitudinal network considering changes in individual components over time.

Given the paucity of studies exploring psychological distress among IVF-ET women from a network analysis perspective, as well as the fact that current state-of-the-art network methods are not confirmatory, we do not propose specific hypotheses for our research questions. However, drawing on previous studies on network analyzes of psychological distress (Beard et al., 2016; Wang et al., 2020) and incorporating the characteristics of psychological distress in Chinese infertility patients (Cao et al., 2022), we may tentatively suggest that (1) there would be positive connections between most of the different symptoms of psychological distress, (2) network structures would be similar over time, and (3) in terms of the patterns of interconnections and network properties (strength centrality and bridge centrality) would change over time, but we did not have specific expectations in this regards.

## Materials and methods

### Setting

A longitudinal study was designed to assess the depressive and anxiety symptoms of women throughout the IVF-ET treatment. Eligible women were recruited at the Reproductive Medicine Center in a public hospital in Shandong Province, China, from March 2019 to May 2020. The eligibility criteria were: being a woman undergoing the first cycle of IVF, consenting to take part in the study, and having no hypertension, diabetes, coronary heart disease, or other major diseases. Exclusion criteria were: not being interested in the study and diagnosed psychiatric disorders. To be specific, women who self-reported a past or current diagnosis of psychiatric illness and/or psychotropic substance use were excluded.

### Participants

A total of 420 IVF-ET women were invited to participate in this study, but 77 (18.3%) of them did not participate in the assessment at baseline, mainly because they refused due to non-interest, or did not have enough time to complete the assessment, or had a history of psychiatric disorders. There were 343 participants who eventually completed the baseline self-report questionnaires on the day of admission (T1) in a private room at the Reproductive Medicine Center. Information on demographics, clinical characteristics, and psychological well-being (depression and anxiety) was collected. Waves two to four were conducted upon the IVF-ET cycle start (T2), on the day of oocyte retrieval (T3) and on the day before the pregnancy test 2 weeks after embryo transfer (T4), respectively. Additionally, participants completed depression and anxiety questionnaires from T2 to T4. At T2, 269 participants followed; at T3, 261 continued participating; and at T4, 212 participants were left. The main reason for missing follow-ups was not filling the questionnaires on time. We adopted a sample size calculation analysis in the context of cross-sectional network models proposed by Constantin et al. (2021), which was implemented by the *powerly* package in R. For a network model consisting of 16 nodes with a sensitivity of 0.6, a probability of 0.8, and a density of 0.2, a sample size of 211 participants was recommended. The cross-sectional network models from T1 to T4 in the present study all met the recommended sample size, although the sample size decreased across the four waves. It should be noted that there is no established method for a formal power analysis available for longitudinal network models, although the sample size was similar to that used in another study of the roughly same model (Miers et al., 2020).

### Measures

#### Demographics and clinical characteristics

The demographic items included questions on age, body mass index (BMI), ethnicity, education level, location, working status, family income (Yuan/monthly), and family type (living with a spouse or not). We assessed clinical characteristics, including duration of infertility and causes of infertility (if male, female, or both, or unexplained).

#### Depression

The Patient Health Questionnaire-9 (PHQ-9) was used to assess depression symptoms from T1 to T4 (Johansson et al., 2021). The scale contains nine items on a four-point scale from 0 (not at all) to 3 (almost every day) and a total score ranging from 0 to 27, with higher scores indicating more severe depression symptoms (Kroenke et al., 2001). A validated study suggested that a cut-off score of 10 or above should be used to identify a participant with depression symptoms (Levis et al., 2019). The PHQ-9 has been shown to be a reliable and valid brief depression assessment tool in infertile women (Evans-Hoeker et al., 2018; Bai et al., 2019a), with Cronbach’s alpha values ranging from 0.874 to 0.912 for the four waves in this study. The items of the PHQ-9 and their reference names are presented in Supplementary Table S1.

#### Anxiety

The Generalized Anxiety Disorder Scale-7 (GAD-7) was used to assess anxiety symptoms from T1 to T4 (Spitzer et al., 2006). The scale has seven questions with scores ranging from 0 (not at all) to 3 (almost every day) for each item, yielding a total score between 0 and 21, with higher scores indicating higher levels of anxiety symptoms. A validated study has suggested that a cut-off score of 10 or above should be used to identify a participant with anxiety symptoms (Spitzer et al., 2006). The GAD-7 has been shown to be a reliable and valid brief anxiety assessment tool for infertile women (Bai et al., 2019a; Barra et al., 2020), with Cronbach’s alpha values ranging from 0.928 to 0.957 for the four waves in this study. The items of the GAD-7 and their reference names are listed in Supplementary Table S1.

### Statistical analysis

Continuous variables are reported as means (standard deviation), and categorical measures are summarized as numbers (percentage). Sample sociodemographic and clinical characteristics were compared between the withdrawal (*n* = 133) and follow-up (*n* = 210) groups using Chi-square tests and independent samples *t-tests*. The significance level was set at 0.05, using SPSS (version 24.0; IBM Corp., Armonk, NY, United States). We then performed the network analysis, including network estimation, network stability, and network differences in R (software).

#### Network estimation

To illustrate the development of the relationship between depression and anxiety symptoms in women undergoing IVF-ET, for each stage, we estimated a least absolute shrinkage and selection operator (LASSO) regularized network that included PHQ-9 and GAD-7 items. Gaussian graphical models with the graphical lasso were constructed using the R software package, *qgraph* (Friedman, 2008). A Gaussian graphical model is an undirected weighted network where edge weights can be interpreted as partial correlation coefficients (i.e., controlling for the effects of all other items in the network; Rouquette et al., 2018). A regularization technique, the LASSO procedure uses a penalized regression method that reduces overfitting by performing shrinkage and model selection simultaneously (Tibshirani, 1996; Epskamp and Fried, 2018). We employed the extended Bayesian information criterion (EBIC) to estimate the optimal value of the penalty parameter using the R package *bootnet* (Foygel and Drton, 2010). As network analysis does not deal with missing data, the available complete data at each stage were used to estimate the network; therefore, *n* varied for each network. To facilitate visual comparisons, all networks were developed using a fixed node placement based on the Fruchterman-Reingold algorithm (Fruchterman and Reingold, 1991; Epskamp et al., 2012).

In addition, we explored which symptoms were most central to the resulting networks by examining strength centrality, the absolute sum of edge weights connected to a node. Previous research indicated that strength centrality is the simplest centrality coefficient (Miers et al., 2020). The advantages of strength centrality include its clear relationship with well-understood psychometric measures and its accessible robust estimates (Epskamp et al., 2016; Haslbeck and Waldorp, 2018). We performed the centrality plot function from the R package *qgraph* for this analysis (Epskamp et al., 2012). We then estimated the bridge centrality indices to identify the symptoms as a bridge between anxiety and depressive symptoms using the bridge function via the R package *networktools* (Jones, 2017). Three indices were investigated: bridge betweenness, bridge closeness, and bridge strength. The only difference between network and bridge centrality is that the two related symptoms come from different symptom communities. The bridge centrality of the nodes accesses the role of a symptom in connecting two mental disorders (Jones et al., 2019). The analysis of checking the stability of the network structure was described in the section titled Supplementary Information.

#### Network differences

To investigate the changes in the networks from T1 to T4, global connectivity (network structure and global strength) and local connectivity (edge weights) for dependent samples were examined. The network structure invariance test investigated whether the structure of the network was identical over time. The global strength invariance test, which represents the overall connectivity (weighted absolute sum of all edges in the network) was the same each time. We used a permutation test called the *Network Comparison Test* (NCT) in the R package to complete this analysis (van Borkulo et al., 2017). A significance level of 0.05 (*p* < 0.05) was applied, and a Bonferroni correction was used for multiple comparisons.

#### Change trajectory network

To explore whether the network changed, we also used longitudinal data to connect the change trajectories on the nodes to each other, as recommended by previous studies (von Klipstein et al., 2018; Miers et al., 2020). Ordinary Least Squares (OLS) regression was performed to estimate the (linear) slope of the score for each node per participant from T1 to T4. Thus, the slope scores refer to the change in each node over time for each individual. All slopes were then used as variables to evaluate the network, in which we set the same parameters as the cross-sectional network (LASSO and EBIC model selection).

## Results

The baseline demographics and clinical characteristics of the participants are presented in Table 1. The difference between the general participant characteristics of those who dropped from this study (*n* = 133) and those of the participants who completed all four assessments (*n* = 210) is shown in Supplementary Table S2. The results showed that there were no significant differences in demographic and clinical information, except for age. The missing participants were mostly older (*t* = 2.593, *p* = 0.01).

**Table 1:** Baseline sociodemographic data, and clinical characteristics of women undergoing IVF-ET (*n* = 343).

Variables	Mean (SD)/*n* (%)
Age, mean (SD), years	30.68 ± 4.10
BMI, mean (SD), kg/m^2^	23.38 ± 3.40
Ethnicity	
Han	339 (98.8%)
Minority	4 (1.2%)
Marital status	
First marriage	291 (84.8%)
Remarriage	52 (15.2%)
Education	
High school or less	186 (54.2%)
College or above	157 (45.8%)
Location	
Rural	99 (28.9%)
Suburban	100 (29.2%)
Urban	144 (42.0%)
Occupation	
Unemployed	87 (25.4%)
Employee	256 (74.6%)
Family income (Yuan/monthly)	
≤4,000 Yuan	168 (49.0%)
>4,000 Yuan	175 (51.0%)
Family type	
Only live with your spouse	273 (79.6%)
Others	70 (20.4%)
Duration of infertility, mean (SD), years	3.47 ± 2.46
Causes for infertility	
Male	50 (14.6%)
Female	202 (58.9%)
Both	23 (6.7%)
Unexplained	68 (19.8%)

IVF-ET, *in vitro* fertilization and embryo transfer; BMI, body mass index.

### Network estimation and comparison

Before characterizing the depression/anxiety symptom network and quantifying the property differences over the course of IVF-ET treatment, we used the bootstrap method to evaluate the stability of the symptom networks. As shown in Supplementary Figures S1, S2, most of the edges and centrality (strength and bridge betweenness) were stable. Detailed results are provided in the Supplementary Information section. Thus, the network differences in this study could reflect a relatively solid change in the pattern of psychological symptom interaction over the course of IVF-ET treatment.

**Figure 1:**
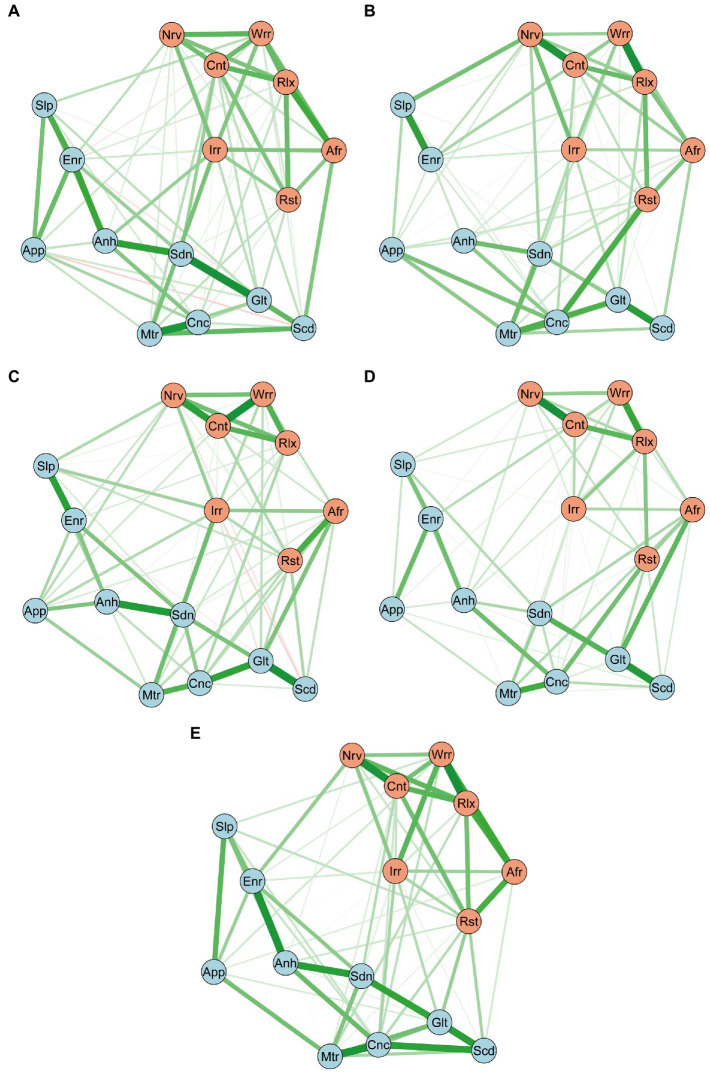
Networks of Depression/anxiety Symptoms at T1 (**A**; *n* = 343), at T2 (**B**; *n* = 269), at T3 (**C**; *n* = 261), at T4 (**D**; *n* = 212), and T1-T4 Slopes Network (**E**; *n* = 210). The blue nodes indicate the PHQ-9 items, and the orange nodes indicate the GAD-7 items. Positive edges appear green, negative red, and stronger and saturated edges represent strong regularized partial correlations. Anh, Anhedonia; Sdn, Sadness; Slp, Sleep; Enr, Energy; App, Appetite; Glt, Guilty; Cnc, Concentration; Mtr, Motor; Scd, Suicide; Nrv, Nervous; Cnt, Control; Wrr, Worry; Rlx, Relax; Rst, Restless; Irr, Irritable; Afr, Afraid; PHQ-9, Patient Health Questionnaire-9; GAD-7, Generalized Anxiety Disorder-7.

Figures 1A–D present the estimated networks at the four time points. The detailed edge weights are shown in Supplementary Tables S3–S6. The network comparison test showed that no global differences were found between network structures and global strengths from T1 to T4: *M* = 0.216–0.275, *p* = 0.26 ~ 0.84, and *S* = 0.036 ~ 0.063, *p* = 0.75 ~ 0.92 (global strength at T1 = 7.48; at T2 = 7.51; at T3 = 7.45; at T4 = 7.51). However, local differences were observed in multiple edges and nodes over time. Locally, over the course of IVF-ET treatment, network differences were found not only in network properties (strength centrality and bridge centrality), but also in symptom connections (edge weights).

**Figure 2:**
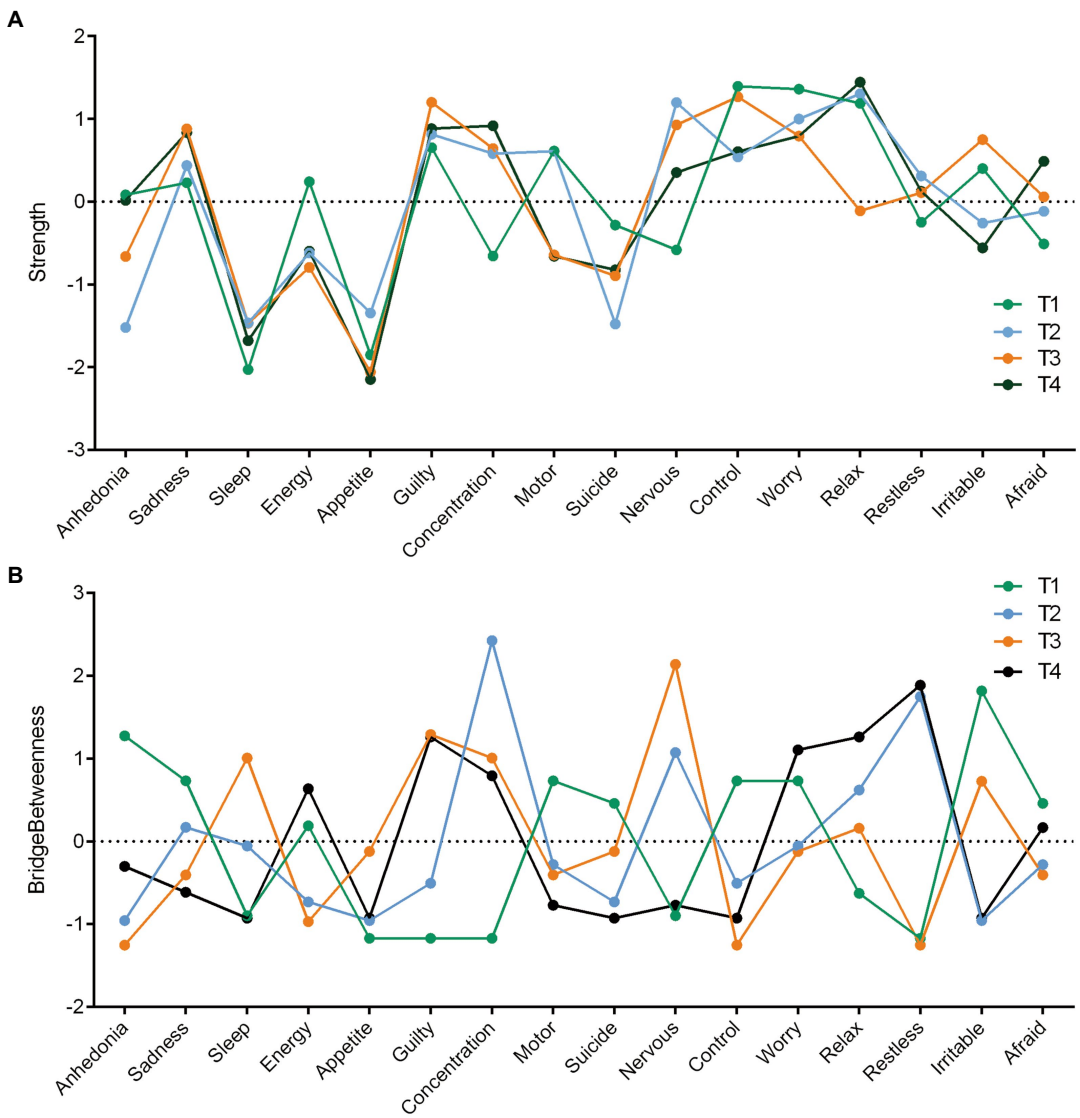
Nodes Strength and Bridge Betweenness Centrality Estimates for the PHQ-9 Items and the GAD-7 Items Completed at T1, T2, T3 and T4. Figure 2 A depicts nodes strength centrality, and Figure 2 B depicts nodes bridge betweenness centrality. PHQ-9: Patient Health Questionnaire-9; GAD-7: Generalized Anxiety Disorder-7.

For the network properties, first, the strength centrality of each symptom in each stage is illustrated in Figure 2A. At T1, “inability to control worry” and “worrying too much,” from the anxiety symptoms, exhibited the highest network strength. During the course of the IVF-ET cycle, although these symptoms decreased, they were still relatively high when compared with other symptoms. Additionally, “sadness” and “guilt” from the depression symptoms exhibited relatively high network strength at T1, and these symptoms increased during the IVF-ET cycle. Second, the bridge centrality (bridge betweenness) of each symptom in each stage is shown in Figure 2B. At T1, the highest was “irritability” from the GAD-7, but it fell sharply after the IVF-ET cycle started. Further, “concentration difficulties,” “nervousness,” and “restlessness” exhibited the highest bridge centrality at T2, T3, and T4, respectively. Of concern, “guilt,” from the PHQ-9, showed the lowest level of bridge centrality at T1, while during the IVF-ET cycle, the symptoms increased and exhibited relatively high bridge betweenness when compared with other symptoms. Importantly, these symptoms may not necessarily be closely related to others. However, they are located in the middle of two mental disorders and may play an important role in mediating the relationship between network symptoms.

In terms of edge weights, as shown in Figures 1A–D, there were some similar patterns of connection in the depression/anxiety symptoms network at different time points, such as a persistent strong connection between “worrying too much” and “inability to relax” within the GAD-7, as well as “concentration difficulties” and “motor symptoms” within the PHQ-9. More notably, significant changes were found in the connections between some network symptoms over time. There were no connections between “nervousness” from the GAD-7 and “sleep difficulties” from the PHQ-9 at T1, whereas a constant and stable connection from T2 to T4 was formed between the two after the patients started the IVF-ET cycle (*p* < 0.001). Additionally, PHQ-9 items “motor symptoms” and “suicide ideation” had a medium degree of connection from T1 to T2, suddenly disappearing at T3, and a weak connection re-emerging at T4 (*p* < 0.05). PHQ-9 items “guilt” and “concentration difficulties” had a consistent connection over the course of the IVF-ET treatment, peaking on the day of the oocyte retrieval (at T3, *p* < 0.05).

### Change trajectory network

The network using the change trajectories of the depression/anxiety components is shown in Figure 1E. The detailed edge weights are shown in Supplementary Table S7. The change trajectory network largely overlapped with the cross-sectional networks (from T1 to T4). Changes in “worrying too much” and “inability to relax” from the GAD-7, as well as “concentration difficulties” and “motor symptoms” from the PHQ-9 were closely related to each other over time. Notably, within the original T1, the suicide ideation symptom was related to guilt in the same symptom community, also exhibiting a cross-community connection with worrying too much in the anxiety symptoms community. Over time, the cross-community connection between suicide ideation and worrying too much disappeared, and the connection with guilt was further strengthened. Thus, changes in suicidal ideation were positively correlated with changes in feeling bad about oneself but not with worrying too much.

## Discussion

The novelty of the present study stems from the application of a network analysis to understand the interactions and changes in anxiety and depressive symptoms during the different phases of the IVF-ET treatment in women. This study identified the central and bridge symptoms, which play a particularly crucial role in different networks at various stages. We found that anxiety-related symptoms may be more central than depression-related symptoms at baseline, but the opposite is true over time; “irritability,” “concentration difficulties,” “nervousness” and “restlessness” showed the highest bridge centrality at T1, T2, T3, and T4, respectively, and “guilt” exhibited increased bridge symptoms over the course of treatment. Furthermore, a constant and stable connection between “nervousness” and “sleep difficulties” was established from T2 onward; over time “suicide ideation” became more closely related to “guilt” but not to “worrying too much.” These findings might be of interest, and may have potential implications for future research at different stages of prevention and intervention efforts. However, these findings need to be interpreted with caution and require future validation, as this may be a chance finding with a limited sample size. In what follows, we discuss these findings in light of strength centrality, bridge centrality, and network connections.

In terms of strength centrality, a previous study in psychiatric patients found that sadness and worry were the most central symptoms in the network of anxiety and depressive symptoms (Beard et al., 2016). In the present study, the inability to control worry and worrying too much from the anxiety symptoms were the most central symptoms in the network at T1. Although the centrality of these symptoms decreased during IVF-ET treatment, they were still relatively high compared to other symptoms. Notably, a relatively high strength centrality of sadness and guilt from the depression symptoms was present at T1 and tended to increase steadily over time, becoming the most central symptoms. Thus, as the IVF-ET cycle started, anxiety symptoms centrality decreased, whereas depression problems were more severe due to the contributions from other non-anxiety-related aspects. These findings are partly inconsistent with the findings that anxiety symptoms are considered more central than depression symptoms in a network estimated from the symptomatology of major depression (Park and Kim, 2020). It also suggests that anxiety-related symptoms and depression-related symptoms may play an important role at different stages of treatment: anxiety-related symptoms of inability to control worry and worrying too much may contribute to the formation of the depression/anxiety symptoms network, while depression-related symptoms, particularly sadness and guilt, although not prominent at baseline, might have a significant role in maintaining the persistence of the depression/anxiety symptoms network by being closely related to other symptoms over the course of treatment. Previous research has indicated that women during IVF-ET treatment are, especially, worried about unsuccessful pregnancy and the fear of loss of control concerning their future without a child (Schaller et al., 2016; Gozuyesil et al., 2020), which may be similar to what is suggested by the central symptoms identified in the data. Researchers have proposed that the inability to reproduce naturally could cause feelings of guilt, which may trigger varying degrees of distress symptoms, potentially explaining why guilt is a central symptom as well as sadness over time (Rooney and Domar, 2018).

When referring to the occurrence of depression and anxiety over the IVF-ET treatment, this current study identified several bridge symptoms at different treatment stages such as irritability, concentration difficulties, nervousness, and restlessness, and that guilt exhibited increased bridge symptoms over time. Only at baseline did irritability show the highest bridge centrality. Some prior studies have established that irritability acts as a bridge mental state which reflects momentary expressions of shared symptoms of depression and anxiety; however, the bridge property of irritability may be less relevant in the context of comorbidity development (Groen et al., 2020). Although the anxiety symptom of nervousness was low at T1, it exhibited the highest bridge centrality at T3. The oocyte retrieval day determined whether the IVF treatment was effective, that is, whether oocytes to continue fertilization are present or not, a situation in which patients often feel nervous about (Miller et al., 2019). This may explain why the nervousness node is the strongest bridge symptom connecting the two mental disorders at T3. Additionally, the symptom of restlessness showed the lowest bridge centrality at T1, being the highest at T4, which is partially in line with previous research. After embryo transfer and before the pregnancy test, patients are always concerned about the cost, whether the treatment will be successful, and what to do if it fails (Liu et al., 2021). A prior study from a sample of patients diagnosed with both depression and anxiety disorders has also suggested that restlessness was a significantly stronger bridge than other symptoms (Kaiser et al., 2021). Similarly, we also identified that, although showing the lowest bridge centrality at T1, concentration difficulties showed the highest bridge effect betweenness at T2, and guilt increased over time. An earlier study indicated that concentration difficulties were a bridge symptom in the general population, but not guilt (Wang et al., 2020). This might further suggest that feelings of guilt are a particularly key symptom link between depression and anxiety disorders among women undergoing IVF-ET treatment, endorsing previous findings from another perspective. Feeling guilt is common among infertile individuals (Rockliff et al., 2014), especially females (Martins et al., 2011). Traditionally, cultural expectations place the need for women to reproduce, and women are easily blamed for pregnancy failure, which could be a powerful psychological stressor triggering depression and anxiety (Wang et al., 2015).

Regarding network connections, the current study found strong links between anxiety symptoms of worrying too much and inability to relax, as well as depression symptoms of concentration difficulties and motor symptoms in the cross-sectional networks from T1 to T4, in line with the trajectory network of changes over time, providing further support for its local consistency. Notably, some other symptoms changed over the course of the IVF-ET treatment. First, a constant and stable connection between nervousness and sleep difficulties from T2 to T4 was found but not at baseline. This finding is in line with the work of Goldstein and colleagues, who found a significant increase in disturbed sleep from the start of ovarian stimulation injections that remained stable over time, possibly relating to psychological distress during IVF (Goldstein et al., 2017). Our findings could elucidate how the sleep and anxiety symptom of nervousness is interrelated, and this might offer a useful modifiable target in sleep management. Our findings tentatively showed how sleep and anxiety symptom of nervousness was interrelated, but require more future research to validate so as to potentially provide a useful and modifiable target for sleep management, which recently was considered an original and innovative parameter in the reproduction field (Caetano et al., 2021). Another interesting finding was that the cross-cluster link between suicide ideation and worrying too much disappeared, whereas the connection with guilt was further strengthened in the change trajectory network. This finding might not be surprising, given the work of Massarotti et al., who found that when the cause of infertility is exclusively female, women experience higher levels of psychological distress across the treatment, which is probably related to a sense of guilt (Massarotti et al., 2019). Additionally, our results showed that the cause of infertility in 58.9% of the participants in this sample was exclusively female which may be consistent with this hypothesis. Given that this symptom of guilt is implicated in suicidal ideation, it may be a key symptom that needs to be considered first and foremost in intervention studies.

Overall, the present study proposed several potential pathways for intervention development to contribute to meeting the mental health needs of women during IVF-ET treatment. In a network at risk, the connections between symptoms are close and powerful, and the activation of one symptom may initiate others, leading to more serious consequences (van Borkulo et al., 2015). Previous studies have recommended that interventions should target symptoms with a high centrality, which may be most closely associated with other symptoms in the network, as these could theoretically reduce the related risk (Levinson et al., 2017; Dobson et al., 2021). Namely, for the treatment of mental health problems associated with women receiving IVF-ET, these findings suggest that researchers and clinicians may be able to pay attention to the symptoms highlighted by our network analysis. For instance, mind–body intervention, cognitive behavioral therapy, and brief mindfulness intervention, targeting the inability to concentrate, inability to relax, and guilt are likely to have the potential to relieve patients from anxiety and depression symptoms (Ying et al., 2016; Bai et al., 2019a).

An advantage of this study is that it is the first network analysis of depression and anxiety in women undergoing IVF-ET treatment utilizing longitudinal, 4-wave data. Importantly, to explore whether the network changed over time, this study investigated the connections between symptoms in a longitudinal network, considering changes in individual components over time. Furthermore, we identify bridge symptoms in networks using a more objective assessment approach, instead of visual inspection or nodes with the highest partial correlations with other nodes (Jones et al., 2019). There were limitations to this study. First, the use of self-report measures entails the risk of report bias. Second, the sample had its own specific cultural context (China) and included people in their first treatment cycle, which may limit the generalizability of the results. Therefore, further research is needed to consolidate these preliminary results among women in various cultures and different treatment cycles. Third, while the recommended cut-off value is 0.25, which is somewhat arbitrary and should not be taken as definite guidelines (Epskamp et al., 2016), the bridge betweenness at T2 and T3, which showed a CS lower than 0.25, should be interpreted with caution. Fourth, although we used a considerably large sample in the baseline, the reduced number of samples in the remaining three waves, especially in change trajectory network model, may affect the reliability of the parameter estimates, which means that the results should be interpreted with caution. This study’s findings need further validation through a larger, representative sample of IVF-ET women. Finally, although the current sample included participants with depression and anxiety scores in the clinical range (at T1, 15.2% of participants scored above the PHQ-9 clinical cut-off of 10, and 12.5% of participants scored above the GAD-7 clinical cut-off of 10), replication of the network in a clinical sample with diagnoses of depressive and anxiety disorders is required.

## Conclusion

This is the first network analysis to focus on psychological distress during IVF-ET treatment in women, which offers promising insights into understanding the interactions and changes in depression and anxiety symptoms. The present study revealed central and bridge symptoms over different stages of IVF-ET treatment aimed at providing clinical recommendations for psychological interventions with the goal of decreasing the co-occurrence of symptoms between various psychological health issues. Further studies should continue to confirm their causal role and to identify their neurological mechanism and cognitive underpinnings in order to improve services for this population.

## Data availability statement

The original contributions presented in the study are included in the article/Supplementary material, further inquiries can be directed to the corresponding author.

## Ethics statement

The study followed the Declaration of Helsinki and was approved by the Ethics Committee of the School of Nursing and Rehabilitation, Shandong University (2019-R-029). All participants volunteered to complete the interviews without any incentive and provided written informed consent. The patients/participants provided their written informed consent to participate in this study. Written informed consent was obtained from the individual(s) for the publication of any potentially identifiable images or data included in this article.

## Author contributions

LW designed the study, performed the analytical experiments, and drafted and revised the manuscript. LS designed the study and collected data. JW, YS, and XZ revised the article for important intellectual content. YH and YL conducted data analysis and revised the article. FC designed the trial, supervised the study, and approved the manuscript. All authors contributed to the article and approved the submitted version.

## Funding

This study was supported by the Surface Project of National Natural Science Foundation of China (Grant Number: 32071084).

## Conflict of interest

The authors declare that the research was conducted in the absence of any commercial or financial relationships that could be construed as a potential conflict of interest.

## Publisher’s note

All claims expressed in this article are solely those of the authors and do not necessarily represent those of their affiliated organizations, or those of the publisher, the editors and the reviewers. Any product that may be evaluated in this article, or claim that may be made by its manufacturer, is not guaranteed or endorsed by the publisher.
