# Supplementary material for: Psychological distress among women undergoing in vitro fertilization-embryo transfer: A cross-sectional and longitudinal network analysis
Source: Front Psychol. 2023 Jan 5;13:1095365. doi: 10.3389/fpsyg.2022.1095365 (PMC9849569; doi:10.3389/fpsyg.2022.1095365)
Supplement: Supplementary file 1 [file Presentation_1.pdf]

Supplementary Information

Journal name:

Frontiers in Psychology

Title:

Psychological Distress among Women Undergoing In Vitro Fertilization-Embryo Transfer: A Cross-Sectional and Longitudinal Network Analysis

Author affiliations:

Liuliu Wu[1†], Lijing Sun[2], Juan Wang[1], Yaoyao Sun[3], Xuan Zhang[1], Yongqi Huang[1], Yan'e Lu[1], Fenglin Cao[1*]

[1]Department of Health Psychology, School of Nursing and Rehabilitation, Cheeloo College of Medicine, Shandong University, No. 44 Wenhua Xi Road, Jinan city, Shandong province, 250012, P.R. China

[2]Center for Reproductive Medicine, Cheeloo College of Medicine, Shandong University, Jinan city, Shandong province, 250012, P.R. China

[3]Institute of Mental Health, Peking University Sixth Hospital, Beijing 100191, China

[*]Corresponding author: Fenglin Cao, Department of Health Psychology, School of Nursing and Rehabilitation, Cheeloo College of Medicine, Shandong University, No. 44 Wenhua Xi Road, Jinan city, Shandong province, 250012, P.R. China. E-mail: fenglin@sdu.edu.cn.

[†]ORCID: 0000-0002-1162-5079

Analysis of Network Stability

A bootstrap approach with 1000 replicates was used to evaluate the stability of edge weights. We plotted the average values and 95% confidence intervals of the edge weights of the networks from the bootstrapped sample with the edge weights of the network from the current sample [1, 2]. When the edge weights of the current sample were largely consistent with the edge weights of the bootstrapped sample, we could consider the network structure to be stable. To further assess the stability of the centralities, we first repeatedly calculated the correlation between centrality indices estimated from the original sample and centrality indices estimated after dropping a certain portion of the cases. If correlation values decreased substantially with a decrease in nodes or participants, this centrality metric would be considered unstable. Second, we determined centrality stability using the case-dropping bootstrap method and calculated the correlation stability (CS) coefficient. CS denotes the maximum proportion of cases that could be eliminated, such that with a 95% probability, the correlation would reach a certain value (0.7, in the current study, cor = 0.7). A $CS_{cor = 0.7}$ above 0.25, but preferentially above 0.5, was recommended [3]. The bootstrap procedure and overall stability for centralities were calculated through *bootnet*.

Results of Network Stability

First, as shown in Fig.S1, a noticeable proportion of the edge weights in the current sample was consistent with the bootstrapped sample, which primarily indicated that the network structures of the current symptom networks were relatively stable. Second, the strength of the network exhibited relatively stable structures at the four time points ($CS_{cor=0.7}$ = 0.265, 0.554, 0.337, 0.481, respectively). In addition, the bridge strength and the bridge closeness of the network were extremely unstable at all time points (for T1, T2, and T3, $CS_{cor=0.7}$ = 0, all). The bridge betweenness of the network exhibited relatively stable structures at T1 and T4 ($CS_{cor=0.7}$ = 0.265 and 0.269, respectively), but the bridge betweenness at T2 and T3 exhibited fewer stable structures, as bridge betweenness both showed a CS lower than 0.25. Therefore, bridge strength and closeness were not included in any further analysis, and any interpretations regarding the bridge betweenness of the symptom network at T2 and T3 should be cautious. Moreover, as displayed in Fig. S2, using 30% of the cases, the average correlations with the original sample of strength and bridge betweenness exceeded 0.25, for all time points, which indicated that centrality indicators for strength and bridge betweenness preserved relatively stable structures over time.

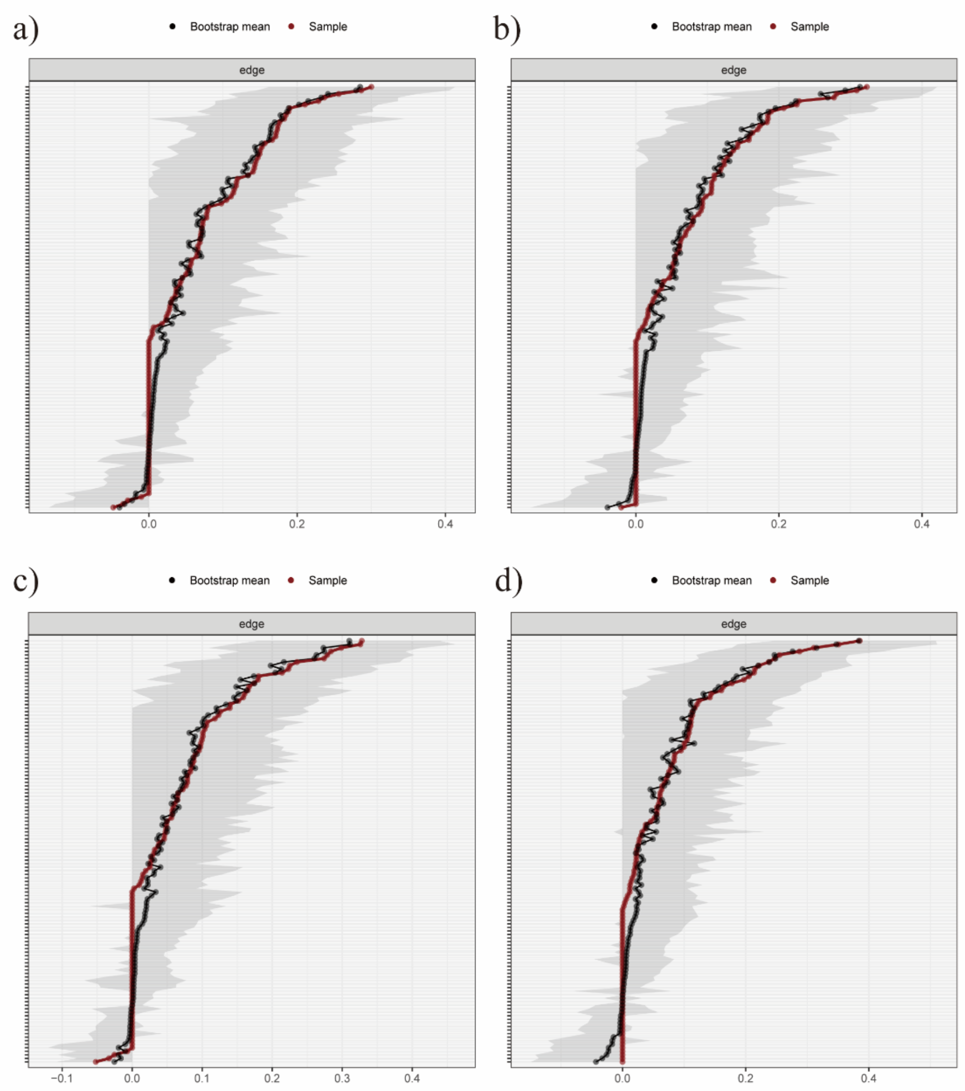

Fig. S1 Stability of Edge Weights. T1: Fig. S1a, T2: Fig. S1b, T3: Fig. S1c, and T4: Figure S1d. The x-axle indicates the edge weights and the y-axle indicates the nodes linked by the edges. The black dots denote the mean value of the bootstrapped edge weights and the red dots denote the edge weights from current sample. The black lines denote the 95% confidence intervals of the bootstrapped sample.

The red dots represent the value (X-axis) of the edge weights (Y-axis). The black dots represent the mean value of the bootstrapped edge weights and the grey lines represent their bootstrapped 95% confidence interval.

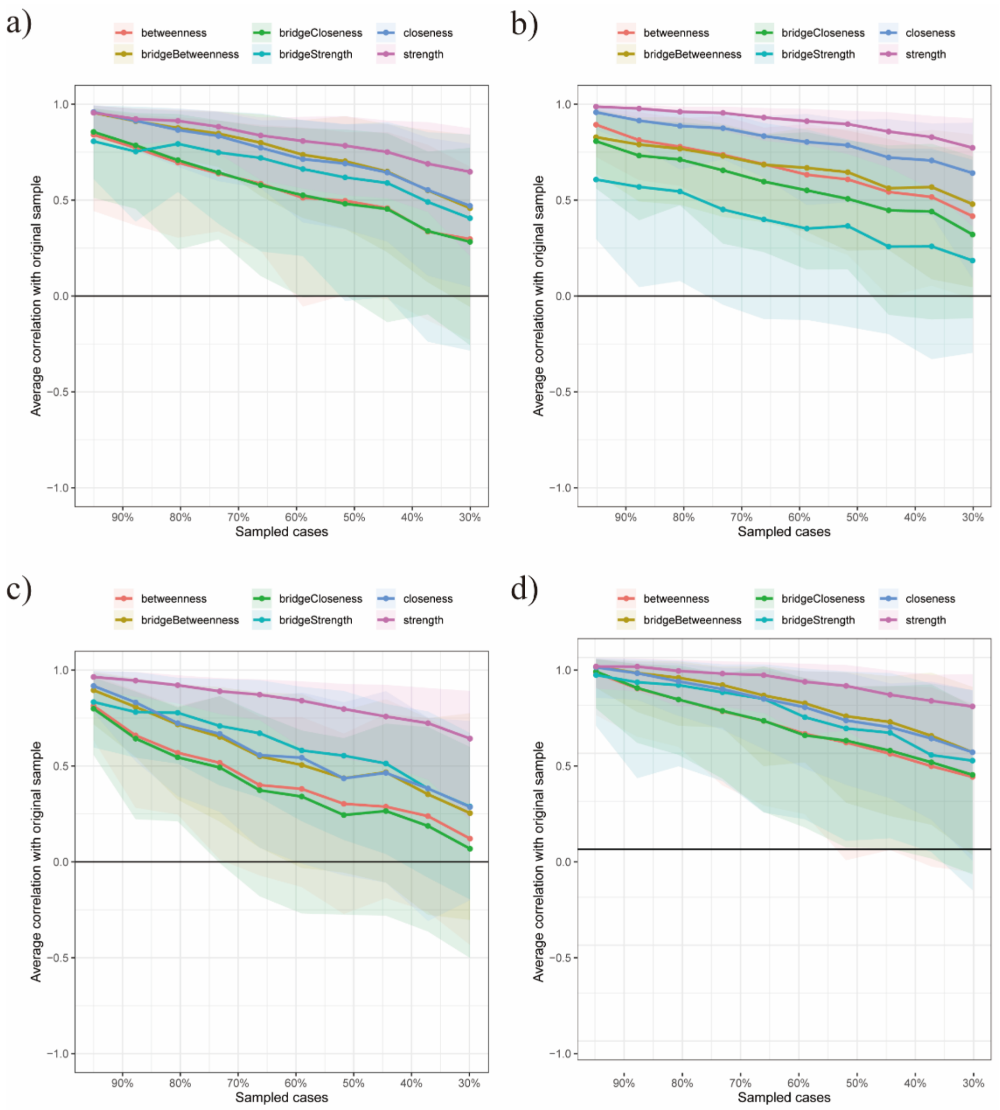

Fig. S2 Stability of network structures. T1: Fig. S1a, T2: Fig. S1b, T3: Fig. S1c, and T4: Fig. S1d. The x-axle denotes the included portion of cases, and the y-axle denotes the correlations between centrality indices estimated from the original sample and centrality indices estimated after dropping a certain portion of the cases. Lines with different colors represent different network properties. The shades indicate the range from the 2.5th quantile to the 97.5th quantile.

Table S1. Questionnaires and Items Used for the Assessment of depression and anxiety symptoms.

	Reference Name	Item
PHQ-9	Anhedonia	Little interest or pleasure in doing things
	Sadness	Feeling down, depressed, or hopeless
	Sleep	Trouble falling or staying asleep, or sleeping too much
	Energy	Feeling tired or having little energy
	Appetite	Poor appetite or overeating
	Guilty	Feeling bad about yourself - or that you are a failure or have let yourself or your family down
	Concentration	Trouble concentrating on things, such as reading the newspaper or watching television
	Motor	Moving or speaking so slowly that other people could have noticed or the opposite - being so fidgety or restless that you have been moving around a lot more than usual
	Suicide	Thoughts that you would be better off dead, or of hurting yourself in some way
GAD-7	Nervous	Feeling nervous, anxious or on edge
	Control	Not being able to stop or control worrying
	Worry	Worrying too much about different things
	Relax	Trouble relaxing
	Restless	Being so restless that it is hard to sit still
	Irritable	Becoming easily annoyed or irritable
	Afraid	Feeling afraid as if something awful might happen

PHQ-9: The Patient Health Questionnaire-9; GAD-7: The Generalised Anxiety Disorder Scale

Table S2. Comparisons in sample sociodemographic and clinical characteristics at baseline between participants who followed up from T1 to T4 and participants who withdrew at follow-up.

Variables	Withdrawal *n* = 133	Retention *n* = 210	t/χ^2	*P* value
Age, mean (*SD*), years	31.39 (4.28)	30.22 (3.92)	2.593	0.01
BMI, mean (*SD*), kg/m^2	23.49 (3.49)	23.31 (3.35)	0.491	0.62
Ethnicity			0.215	0.64
Han	131 (98.5%)	208 (99.0%)		
Minority	2 (1.5%)	2 (1.0%)		
Marital status			0.768	0.38
First marriage	110 (82.7%)	181 (86.2%)		
Remarriage	23 (17.3%)	29 (13.8%)		
Education			1.177	0.28
High school or less	56 (42.1%)	101 (48.1%)		
College or above	77 (57.9%)	109 (51.9%)		
Location			0.244	0.89
Rural	40 (30.1%)	59 (28.1%)		
Suburban	37 (27.8%)	63 (30.0%)		
Urban	56 (42.1%)	88 (41.9%)		
Occupation			0.035	0.85
Unemployed	33 (24.8%)	54 (25.7%)		
Employee	100 (75.2%)	156 (74.3%)		
Family income (Yuan/monthly)			0.731	0.39
≤4000 Yuan	69 (51.9%)	99 (47.1%)		
>4000 Yuan	64 (48.1%)	111 (52.9%)		
Family type			0.261	0.61
Only live with your spouse	104 (78.2%)	169 (80.5%)		
Others	29 (21.8%)	41 (19.5%)		
Duration of infertility, mean (SD), years	3.46 (2.61)	3.47 (2.37)	0.035	0.97
Causes for infertility			5.143	0.16
Male	12 (9.0%)	38 (18.1%)		
Female	82 (61.7%)	120 (57.1%)		
Both	7 (5.3%)	16 (7.6%)		
Unexplained	32 (24.0%)	36 (17.1%)		

Table S3. Estimated Edge Weights at T1

	Anhedonia	Sadness	Sleep	Energy	Appetite	Guilty	Concentration	Motor	Suicide	Nervous	Control	Worry	Relax	Restless	Irritable	Afraid
Anhedonia	0.00	0.26	0.10	0.24	0.07	0.00	0.14	0.00	0.00	0.04	0.00	0.00	0.00	0.00	0.11	0.00
Sadness	0.26	0.00	0.00	0.12	0.00	0.30	0.00	0.00	0.12	0.03	0.12	0.00	0.01	0.03	0.00	0.00
Sleep	0.10	0.00	0.00	0.21	0.16	0.07	0.05	0.00	0.00	0.00	-0.01	0.08	0.00	0.02	0.00	0.00
Energy	0.24	0.12	0.21	0.00	0.17	0.00	0.00	0.00	-0.03	0.07	-0.03	0.00	0.04	0.00	0.05	0.00
Appetite	0.07	0.00	0.16	0.17	0.00	0.06	0.10	0.07	-0.05	0.00	0.00	0.06	0.00	0.00	0.00	0.00
Guilty	0.00	0.30	0.07	0.00	0.06	0.00	0.08	0.11	0.18	0.00	0.07	0.00	0.00	0.07	0.08	0.00
Concentration	0.14	0.00	0.05	0.00	0.10	0.08	0.00	0.29	0.06	0.00	0.00	0.03	0.07	0.04	0.02	0.00
Motor	0.00	0.00	0.00	0.00	0.07	0.11	0.29	0.00	0.18	0.05	0.08	0.00	0.03	0.07	0.15	0.00
Suicide	0.00	0.12	0.00	-0.03	-0.05	0.18	0.06	0.18	0.00	0.00	0.07	0.04	0.00	0.03	0.00	0.14
Nervous	0.04	0.03	0.00	0.07	0.00	0.00	0.00	0.05	0.00	0.00	0.17	0.19	0.15	0.04	0.15	0.00
Control	0.00	0.12	-0.01	-0.03	0.00	0.07	0.00	0.08	0.07	0.17	0.00	0.17	0.19	0.15	0.00	0.07
Worry	0.00	0.00	0.08	0.00	0.06	0.00	0.03	0.00	0.04	0.19	0.17	0.00	0.18	0.05	0.14	0.17
Relax	0.00	0.01	0.00	0.04	0.00	0.00	0.07	0.03	0.00	0.15	0.19	0.18	0.00	0.17	0.02	0.23
Restless	0.00	0.03	0.02	0.00	0.00	0.07	0.04	0.07	0.03	0.04	0.15	0.05	0.17	0.00	0.12	0.13
Irritable	0.11	0.00	0.00	0.05	0.00	0.08	0.02	0.15	0.00	0.15	0.00	0.14	0.02	0.12	0.00	0.14
Afraid	0.00	0.00	0.00	0.00	0.00	0.00	0.00	0.00	0.14	0.00	0.07	0.17	0.23	0.13	0.14	0.00

Table S4. Estimated Edge Weights at T2

	Anhedonia	Sadness	Sleep	Energy	Appetite	Guilty	Concentration	Motor	Suicide	Nervous	Control	Worry	Relax	Restless	Irritable	Afraid
Anhedonia	0.00	0.18	0.00	0.07	0.06	0.02	0.14	0.00	0.00	0.00	0.00	0.03	0.00	0.00	0.05	0.05
Sadness	0.18	0.00	0.00	0.00	0.00	0.14	0.00	0.18	0.11	0.11	0.00	0.07	0.02	0.08	0.05	0.05
Sleep	0.00	0.00	0.00	0.28	0.10	0.00	0.04	0.00	0.00	0.17	0.01	0.02	0.00	0.02	0.00	0.00
Energy	0.07	0.00	0.28	0.00	0.05	-0.02	0.05	0.00	0.00	0.08	0.01	0.11	0.06	0.00	0.06	0.00
Appetite	0.06	0.00	0.10	0.05	0.00	0.00	0.16	0.14	0.00	0.05	0.00	0.00	0.01	0.06	0.00	0.00
Guilty	0.02	0.14	0.00	-0.02	0.00	0.00	0.09	0.22	0.28	0.00	0.02	0.04	0.09	0.03	0.09	0.01
Concentration	0.14	0.00	0.04	0.05	0.16	0.09	0.00	0.21	0.00	0.00	0.09	0.00	0.00	0.23	0.00	0.00
Motor	0.00	0.18	0.00	0.00	0.14	0.22	0.21	0.00	0.11	0.00	0.00	0.00	0.00	0.06	0.09	0.00
Suicide	0.00	0.11	0.00	0.00	0.00	0.28	0.00	0.11	0.00	0.00	0.02	0.00	0.00	0.00	0.00	0.11
Nervous	0.00	0.11	0.17	0.08	0.05	0.00	0.00	0.00	0.00	0.00	0.31	0.12	0.12	0.00	0.17	0.00
Control	0.00	0.00	0.01	0.01	0.00	0.02	0.09	0.00	0.02	0.31	0.00	0.13	0.19	0.08	0.04	0.12
Worry	0.03	0.07	0.02	0.11	0.00	0.04	0.00	0.00	0.00	0.12	0.13	0.00	0.32	0.00	0.13	0.12
Relax	0.00	0.02	0.00	0.06	0.01	0.09	0.00	0.00	0.00	0.12	0.19	0.32	0.00	0.18	0.00	0.16
Restless	0.00	0.08	0.02	0.00	0.06	0.03	0.23	0.06	0.00	0.00	0.08	0.00	0.18	0.00	0.07	0.16
Irritable	0.05	0.05	0.00	0.06	0.00	0.09	0.00	0.09	0.00	0.17	0.04	0.13	0.00	0.07	0.00	0.11
Afraid	0.05	0.05	0.00	0.00	0.00	0.01	0.00	0.00	0.11	0.00	0.12	0.12	0.16	0.16	0.11	0.00

Table S5. Estimated Edge Weights at T3

	Anhedonia	Sadness	Sleep	Energy	Appetite	Guilty	Concentration	Motor	Suicide	Nervous	Control	Worry	Relax	Restless	Irritable	Afraid
Anhedonia	0.00	0.30	0.11	0.15	0.16	0.00	0.08	0.06	0.00	0.00	0.00	0.00	0.00	0.00	0.02	0.00
Sadness	0.30	0.00	0.05	0.16	0.00	0.08	0.14	0.07	0.17	0.00	0.03	0.00	0.02	0.03	0.00	0.05
Sleep	0.11	0.05	0.00	0.28	0.00	0.00	0.00	0.00	0.00	0.10	0.00	0.03	0.01	0.07	0.12	0.00
Energy	0.15	0.16	0.28	0.00	0.11	0.00	0.00	0.00	-0.03	0.06	0.03	0.00	0.05	0.00	0.00	0.00
Appetite	0.16	0.00	0.00	0.11	0.00	0.00	0.01	0.12	0.00	0.09	0.06	0.00	0.00	0.00	0.10	0.05
Guilty	0.00	0.08	0.00	0.00	0.00	0.00	0.27	0.06	0.33	0.00	0.00	0.08	0.00	0.09	0.05	0.17
Concentration	0.08	0.14	0.00	0.00	0.01	0.27	0.00	0.23	0.05	0.00	0.00	0.00	0.10	0.09	0.00	0.08
Motor	0.06	0.07	0.00	0.00	0.12	0.06	0.23	0.00	0.00	0.00	0.00	0.01	0.06	0.09	0.18	0.00
Suicide	0.00	0.17	0.00	-0.03	0.00	0.33	0.05	0.00	0.00	-0.03	0.00	-0.01	0.00	0.10	-0.05	0.08
Nervous	0.00	0.00	0.10	0.06	0.09	0.00	0.00	0.00	-0.03	0.00	0.28	0.17	0.18	0.04	0.13	0.00
Control	0.00	0.03	0.00	0.03	0.06	0.00	0.00	0.00	0.00	0.28	0.00	0.33	0.21	0.10	0.03	0.06
Worry	0.00	0.00	0.03	0.00	0.00	0.08	0.00	0.01	-0.01	0.17	0.33	0.00	0.22	0.04	0.10	0.08
Relax	0.00	0.02	0.01	0.05	0.00	0.00	0.10	0.06	0.00	0.18	0.21	0.22	0.00	0.00	0.06	0.03
Restless	0.00	0.03	0.07	0.00	0.00	0.09	0.09	0.09	0.10	0.04	0.10	0.04	0.00	0.00	0.10	0.24
Irritable	0.02	0.00	0.12	0.00	0.10	0.05	0.00	0.18	-0.05	0.13	0.03	0.10	0.06	0.10	0.00	0.14
Afraid	0.00	0.05	0.00	0.00	0.05	0.17	0.08	0.00	0.08	0.00	0.06	0.08	0.03	0.24	0.14	0.00

Table S6. Estimated Edge Weights at T4

	Anhedonia	Sadness	Sleep	Energy	Appetite	Guilty	Concentration	Motor	Suicide	Nervous	Control	Worry	Relax	Restless	Irritable	Afraid
Anhedonia	0.00	0.12	0.08	0.21	0.06	0.03	0.21	0.00	0.00	0.07	0.00	0.07	0.06	0.00	0.00	0.00
Sadness	0.12	0.00	0.11	0.02	0.00	0.25	0.10	0.14	0.10	0.01	0.01	0.00	0.00	0.03	0.06	0.12
Sleep	0.08	0.11	0.00	0.20	0.08	0.00	0.00	0.00	0.00	0.07	0.06	0.00	0.00	0.00	0.02	0.00
Energy	0.21	0.02	0.20	0.00	0.22	0.00	0.00	0.02	0.00	0.02	0.01	0.10	0.00	0.00	0.00	0.00
Appetite	0.06	0.00	0.08	0.22	0.00	0.00	0.00	0.08	0.05	0.00	0.00	0.04	0.00	0.00	0.00	0.00
Guilty	0.03	0.25	0.00	0.00	0.00	0.00	0.06	0.02	0.35	0.00	0.00	0.00	0.00	0.11	0.02	0.24
Concentration	0.21	0.10	0.00	0.00	0.00	0.06	0.00	0.29	0.11	0.00	0.03	0.01	0.06	0.21	0.02	0.00
Motor	0.00	0.14	0.00	0.02	0.08	0.02	0.29	0.00	0.03	0.00	0.02	0.02	0.00	0.00	0.06	0.11
Suicide	0.00	0.10	0.00	0.00	0.05	0.35	0.11	0.03	0.00	0.00	0.00	0.00	0.00	0.08	0.00	0.06
Nervous	0.07	0.01	0.07	0.02	0.00	0.00	0.00	0.00	0.00	0.00	0.39	0.16	0.11	0.08	0.05	0.01
Control	0.00	0.01	0.06	0.01	0.00	0.00	0.03	0.02	0.00	0.39	0.00	0.11	0.25	0.01	0.11	0.04
Worry	0.07	0.00	0.00	0.10	0.04	0.00	0.01	0.02	0.00	0.16	0.11	0.00	0.31	0.02	0.10	0.11
Relax	0.06	0.00	0.00	0.00	0.00	0.00	0.06	0.00	0.00	0.11	0.25	0.31	0.00	0.18	0.16	0.06
Restless	0.00	0.03	0.00	0.00	0.00	0.11	0.21	0.00	0.08	0.08	0.01	0.02	0.18	0.00	0.08	0.14
Irritable	0.00	0.06	0.02	0.00	0.00	0.02	0.02	0.06	0.00	0.05	0.11	0.10	0.16	0.08	0.00	0.12
Afraid	0.00	0.12	0.00	0.00	0.00	0.24	0.00	0.11	0.06	0.01	0.04	0.11	0.06	0.14	0.12	0.00

Table S7. Estimated Edge Weights for T1-T4 slopes network

	Anhedonia	Sadness	Sleep	Energy	Appetite	Guilty	Concentration	Motor	Suicide	Nervous	Control	Worry	Relax	Restless	Irritable	Afraid
Anhedonia	0.00	0.23	0.12	0.24	0.00	0.02	0.15	0.00	0.00	0.03	0.00	0.00	0.00	0.05	0.01	0.00
Sadness	0.23	0.00	0.00	0.11	0.00	0.21	0.00	0.13	0.07	0.00	0.07	0.00	0.00	0.01	0.03	0.00
Sleep	0.12	0.00	0.00	0.13	0.17	0.00	0.05	0.00	0.00	0.00	0.00	0.05	0.00	0.06	0.00	0.00
Energy	0.24	0.11	0.13	0.00	0.10	0.00	0.06	0.01	0.00	0.10	0.00	0.03	0.00	0.00	0.04	0.00
Appetite	0.00	0.00	0.17	0.10	0.00	0.04	0.00	0.14	0.00	0.00	0.00	0.05	0.00	0.00	0.00	0.03
Guilty	0.02	0.21	0.00	0.00	0.04	0.00	0.10	0.14	0.23	0.00	0.00	0.00	0.00	0.10	0.07	0.00
Concentration	0.15	0.00	0.05	0.06	0.00	0.10	0.00	0.24	0.22	0.00	0.05	0.06	0.00	0.07	0.09	0.00
Motor	0.00	0.13	0.00	0.01	0.14	0.14	0.24	0.00	0.09	0.00	0.03	0.00	0.06	0.00	0.01	0.02
Suicide	0.00	0.07	0.00	0.00	0.00	0.23	0.22	0.09	0.00	0.00	0.00	0.00	0.00	0.09	0.02	0.05
Nervous	0.03	0.00	0.00	0.10	0.00	0.00	0.00	0.00	0.00	0.00	0.25	0.12	0.16	0.05	0.11	0.00
Control	0.00	0.07	0.00	0.00	0.00	0.00	0.05	0.03	0.00	0.25	0.00	0.13	0.17	0.14	0.06	0.00
Worry	0.00	0.00	0.05	0.03	0.05	0.00	0.06	0.00	0.00	0.12	0.13	0.00	0.26	0.00	0.16	0.20
Relax	0.00	0.00	0.00	0.00	0.00	0.00	0.00	0.06	0.00	0.16	0.17	0.26	0.00	0.14	0.05	0.19
Restless	0.05	0.01	0.06	0.00	0.00	0.10	0.07	0.00	0.09	0.05	0.14	0.00	0.14	0.00	0.08	0.19
Irritable	0.01	0.03	0.00	0.04	0.00	0.07	0.09	0.01	0.02	0.11	0.06	0.16	0.05	0.08	0.00	0.10
Afraid	0.00	0.00	0.00	0.00	0.03	0.00	0.00	0.02	0.05	0.00	0.00	0.20	0.19	0.19	0.10	0.00

References

[1] Joshua, Curtiss, Masaya, et al. Longitudinal Network Stability of the Functional Impairment of Anxiety and Depression [J]. 2018, 6(3): 325-34.

[2] Wang Y, Hu Z, Feng Y, et al. Changes in network centrality of psychopathology symptoms between the COVID-19 outbreak and after peak [J]. Mol Psychiatry, 2020, 25(12): 3140-9. https://doi.org/10.1038/s41380-020-00881-6

[3] Epskamp S, Borsboom D, Fried E I J B R M. Estimating Psychological Networks and their Accuracy: a Tutorial Paper [J]. 2016, 50(4).
